# Microsurgical Reconstruction of Large, Locally Advanced Cutaneous Malignancy of the Head and Neck

**DOI:** 10.1155/2011/415219

**Published:** 2011-10-20

**Authors:** Joseph L. Hill, Brian Rinker

**Affiliations:** Division of Plastic Surgery, Department of Surgery, University of Kentucky College of Medicine, Lexington, KY 40536-0284, USA

## Abstract

Large, locally advanced cutaneous malignancy of the head and neck region is rare. However, when present, they impart a significant reconstructive challenge. These cancers have a tendency to invade peripheral tissues covering a large surface area as well as expose deeper structures such as skull, dura, orbit, and sinus after resection. Complicating the reconstructive dilemma is the high incidence of individuals who have undergone previous surgery in the region as well as adjuvant radiation therapy, which may preclude the use of local flaps or skin graft. Free tissue transfer provides a reconstructive surgeon the ability to provide well-vascularized tissue with adequate volume not limited by arc of rotation.

## 1. Background

Skin cancer is the most common type of cancer in fair skinned individuals [1]. Basal cell carcinoma is the most common type of skin cancer, affecting approximately 2 million Americans per year [2]. Basal cell carcinoma is followed closely in incidence by squamous cell carcinoma, which accounts for 20% of all skin cancers, with approximately 700,000 new cases identified per year [2, 3]. Basal cell and squamous cell cancers are more common in sun-exposed areas of the body, including the head and neck region [3, 4]. Other less common types of cutaneous malignancy in the head and neck region include melanoma, Merkel cell carcinoma, sebaceous carcinoma, eccrine carcinoma, and dermatofibrosarcoma protuberans. 

The head and neck region is a well-visualized region of the body. Skin cancers in this region are usually easily identifiable with patients typically presenting early in the clinical course of the disease [5]. These skin cancers are amenable to simple resection followed by reconstruction with a skin graft, local flap, or healing by secondary intention [5, 6]. Most patients heal uneventfully with good restoration of function and appearance [5, 6]. Occasionally, however, patients with skin cancers present much later in the clinical course of the disease [7]. These types of cancers have been described as “advanced,” “massive,” “complex,” “gigantic,” and “horrifying [7].” The main reasons that patients present with such extensive tumors are failure of primary treatment and patient neglect [7].

## 2. Reconstructive Dilemma

Fortunately, these types of advanced skin cancers are rare [6, 8]. As an example, the incidence of giant basal cell carcinomas (>5 cm diameter) is less than 1% of all basal cell carcinomas [8]. Despite their infrequent presentation, defects following resection of large cutaneous malignancies present a marked reconstructive challenge [9]. These cancers have a tendency to invade peripheral tissues covering a large surface area as well as invade deeper structures such as skull, dura, orbit, and sinus [7]. Complicating the reconstructive dilemma is the high incidence of individuals who have undergone previous surgery in the region as well as adjuvant radiation therapy, which may preclude the use of local flaps or skin grafts [5]. Moreover, regional flaps often lack adequate volume to reconstruct large defects and are limited by their arc of rotation [7, 10]. As a result, large, locally advanced cancers of the head and neck region were once considered nonoperable secondary to a lack of reconstructive options [7, 10]. The advent of microsurgical free tissue transfer changed the management of these advanced cutaneous malignancies allowing for complete resection of tumor without compromise of tumor margin [5, 10]. Free tissue transfer provides well-vascularized tissue with excellent volume for reconstruction of complex defects of the head and neck region [10].

## 3. Flap Selection

Flap selection is an important component in planning a successful head and neck reconstruction after tumor ablation. Defects in the head and neck can be classified into six anatomical subareas for reconstructive considerations: intraoral, mandibular, midfacial, cranial, cutaneous, and scalp [10]. Upon completion of the resection, the location, the size, the tissue components (skin, soft tissue, or bone) excised, and the compartments (maxilla, orbit, cranium, and mandible) involved are noted [10]. After this analysis, a suitable flap can be selected. 

Unfortunately, it is difficult for an individual surgeon to be comfortable with all of the potential free flaps available for use in the head and neck [10]. As a result, numerous authors have developed algorithms which simplify flap selection [11]. Jones et al. identified seven free flaps suitable for head and neck reconstruction. These flaps are jejunum, radial forearm, rectus abdominis, latissimus dorsi, scapula/parascapular, fibula, and iliac crest [11]. Disa et al. refined this algorithm to only include the radial forearm, fibula, myocutaneous rectus abdominus, and jejunum [12]. 

 Wong and Wei had refined this algorithm further in head and neck reconstruction to include the anterolateral thigh (ALT) flap, radial forearm, jejunum, and fibula [10]. According to Wei, these flaps were chosen because they provide a long vascular pedicle with adequate caliber and contain variable types of tissue. The ALT flap, for example, has become the workhorse flap for soft-tissue reconstruction for this group and can, therefore, be used in the reconstruction of several subareas in the head and neck region. The ALT flap is based on the descending branch of the lateral circumflex femoral artery. The pedicle length has been documented as being up to 18 cm long. The flap can contain vastus lateralis muscle for added bulk, tensor fascia lata for strength, or can be thinned to skin and subcutaneous fat [10]. The flap can be de-epithelialized and used to fill volume and can also be made into a sensate flap via the anterior branch of the lateral cutaneous nerve of the thigh [10]. Perhaps more importantly, however, donor site morbidity is kept to a minimum after harvest of an ALT flap and does not require patient repositioning as is the case when utilizing a similar type of flap for reconstruction like the parascapular flap [11].

## 4. Craniectomy

**Figure 1:**
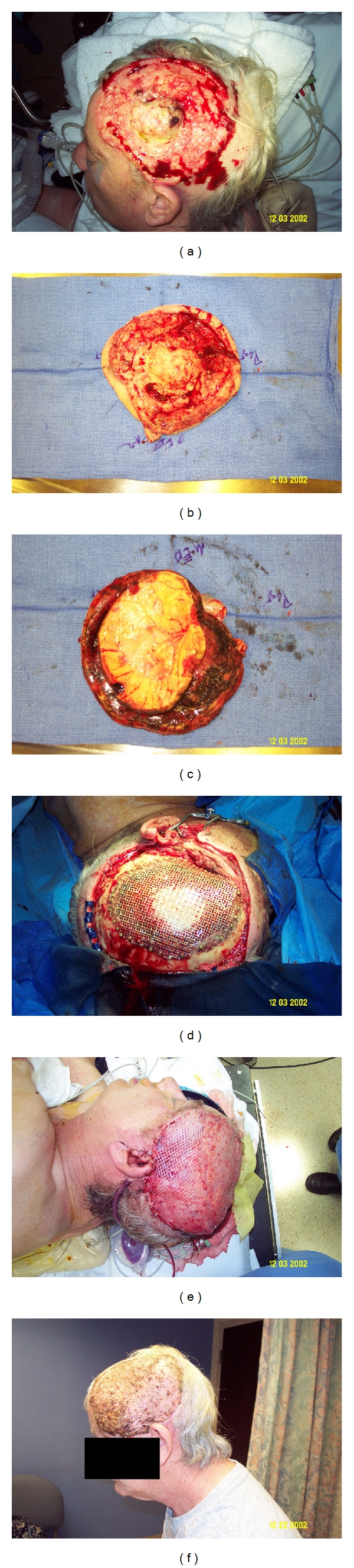
A 65-year-old male with a large, locally advanced left scalp squamous cell carcinoma. (a) Left scalp lesion; (b) excised lesion scalp side; (c) excised lesion cranial side showing parietal cranium; (d) titanium mesh cranioplasty; (e) inset-free latissimus muscle flap with split thickness skin graft; (f) 1-month followup.

Defects in the cranial vault are not uncommon after excision of large, locally advanced cutaneous malignancies of the scalp and forehead. In doing so, underlying dura or brain parenchyma become exposed, which, at the very least, requires soft-tissue coverage. Due to the size of these resections and the limited amount of healthy tissue from local and regional sources, free tissue transfer is necessary [9]. Muscle flaps which are commonly used for scalp or forehead reconstruction after large tumor ablation include the latissimus dorsi and rectus abdominus muscle flaps or latissimus dorsi and rectus abdominus myocutaneous flaps [9]; see Figures 1(a)–1(f). Fasciocutaneous flaps, which have been described for use after these types of resections, include the ALT flap, scapular/parascapular flaps, and radial forearm flap [9]. Each of these flaps can cover large surface areas and have long vascular pedicles [9]. 

It should be noted, however, that craniectomy for any reason, including tumor ablation, is not without complication. Known complications include brain herniation, subdural effusion, syndrome of trephined (ST), infection, hematoma, hydrocephalus, and cerebrospinal fluid leak [13]. ST is a known complication of craniectomy consisting of severe headache, dizziness, undue fatigability, poor memory, irritability, convulsions, mental depression, and intolerance to vibration [14]. 

In a study by Yang et al. [13], 108 patients who suffered closed traumatic brain injury ultimately requiring decompressive craniectomy were retrospectively reviewed. Complications occurred in 54 of the 108 patients. More than one complication occurred in 25.9%. Herniation of parenchyma through the cranial bone defect was found in 27.8% of patients, which commonly leads to venous infarction. This figure included seven out of eighteen patients with small craniectomy defects, thus implicating the dimensions of the craniectomy as a contributing factor to brain herniation [13]. 

In Stiver's review of the literature, increased brain swelling is common following decompressive craniectomy [15]. Brain swelling results from hyperperfusion in the adjacent brain parenchyma as well as loss of resistance in brain tissue lacking a protective skull. This loss of resistance invokes a higher hydrostatic pressure gradient that may permit transcapillary leakage of edema fluid. While these two physiological sequelae of craniectomy are documented to occur following decompressive craniectomy, one could reasonably assume the loss of resistance in brain tissue lacking a protective skull also occurs following craniectomy for other reasons and, therefore, could contribute to brain herniation through a cranial bone defect following tumor ablation [15].

## 5. Cranioplasty

Cranioplasty is utilized to prevent some of the long term sequelae of craniectomy. Indications for cranioplasty according to Lee et al. is to protect the cerebrum and for cosmetic purposes [16]. More recently, many authors believe ST is an indication for cranioplasty [14]. 

Materials available for cranioplasty fall into two categories: autologous or alloplastic. Autogenous bone sources include split calvarial bone graft, iliac crest, and rib. Autogenous bone has been advocated by some secondary to its ability to become incorporated as living tissue and, therefore, has an improved ability to resist infection [16]. Disadvantages of autogenous bone include potential donor site morbidity and increased length of time for harvest [17]. 

Examples of alloplastic materials include titanium mesh, hydroxyapatite, methyl methacrylate, and porous polyethylene [17]; see Figure 1(d). Alloplastic materials have the advantage of being in abundant supply and have no donor site morbidity. However, they are contraindicated in compromised or infected wound beds [16]. 

Cranioplasty is not without its own set of complications. These complications include infection, epidural or subdural fluid collection, seizures, and fixed nenrological deficits [18].

## 6. Orbital Exenteration

Another consideration after ablation of large cutaneous malignancy in the head and neck region is reconstruction options following orbital exenteration. Orbital exenteration involves the removal of orbital contents including the globe, extraocular muscles, periorbital soft-tissue, and varying portions of the orbit. It is usually undertaken for orbital and periorbital malignancies including basal cell and squamous cell carcinoma. 

**Figure 2:**
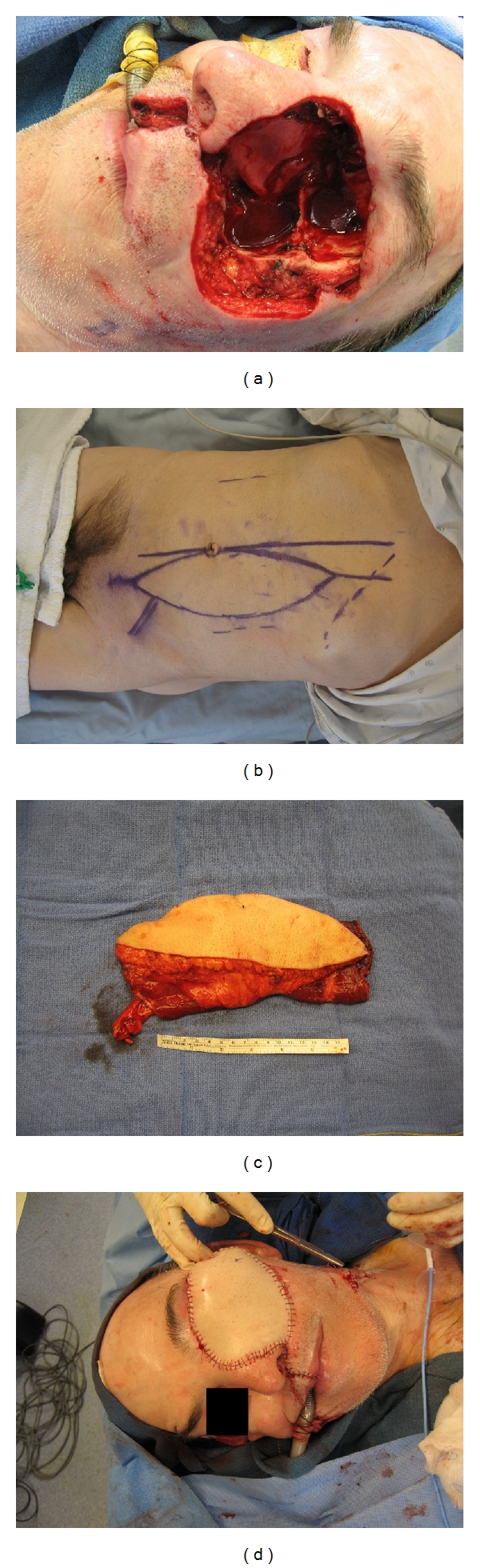
A 56-year-old male with a locally invasive left facial basal cell carcinoma. (a) Maxillectomy plus orbital exenteration; (b), (c) vertical rectus abdominus myocutaneous (VRAM) flap; (d) inset-free VRAM.

The primary goal of reconstruction is to line or fill the orbit with durable tissue that excludes the nasal cavity, paranasal sinuses, and dura. The reconstruction may need to be able to withstand the harmful effects of radiation and to accommodate a prosthesis. Options for reconstruction include split thickness skin graft, full thickness skin graft, regional flap, and free flap depending on the tissue components that remain or are exposed following orbital exenteration. Free flaps which have been documented to be utilized in reconstruction following orbital exenteration include rectus abdominus muscle flap, split thickness skin graft, rectus abdominus myocutaneous flap, and the anterolateral thigh flap [19]; see Figures 2(a)–2(d). 

According to Hanasono et al. [19], selection of the most suitable reconstructive option depends on several factors, including the extent of the resection, the need for adjuvant radiation, and the desire for a prosthesis. The extent of the resection ranges from globe and soft tissue only to globe, soft tissue, bony orbit, and finally, to include all of the above plus the maxilla. Skin grafting should only be utilized for limited resection, no adjuvant radiation therapy, and patient desire for a prosthesis. The need for a free flap is determined by the extent of the resection such that orbital exenteration with a maxillectomy requires free flap reconstruction [19].

## 7. Maxillectomy

**Figure 3:**
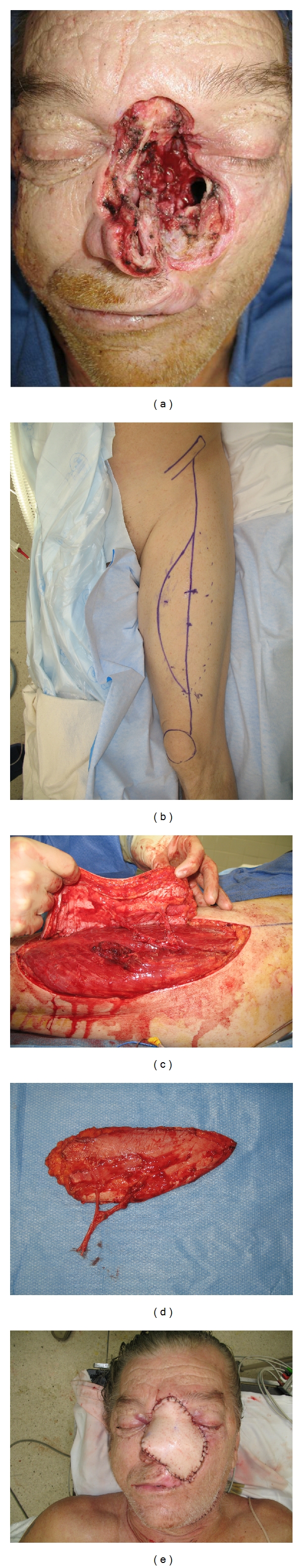
A 61-year-old male with a poorly controlled left facial basal cell carcinoma. (a) Maxillectomy defect; (b), (c), (d) ALT-free flap with long vascular pedicle; (e) inset of ALT-free flap.

Lastly, cutaneous malignancies sometimes extend into the maxilla and nasal cavity necessitating maxillectomy. As indicated by Wells and Luce, these resections are more common with primary sinus malignancy [20]. Nonetheless, the need for reconstructing the maxilla can be an issue following resection of large, locally advanced cutaneous malignancies. Reconstructive goals include wound closure, the restoration of the barrier between the sinonasal cavity and the anterior cranial fossa, the separation of the oral and sinonasal cavities, support of orbital contents, maintenance of ocular globe position, oral continence, speech, mastication, avoidance of ectropion, maintenance of a patent nasal airway, and lastly, facial appearance [21]. Maxillary defects range from limited maxillectomy to total maxillectomy with orbital exenteration [21]. Reconstructive options include free radial forearm flap fasciocutaneous flap, ALT flap, and vertical rectus myocutaneous flap with or without bone grafting depending on the degree of resection [21]; see Figures 3(a)–3(e).

## 8. Summary

Large, locally advanced cutaneous malignancy of the head and neck generally occurs secondary to patient neglect and because of a failure of primary treatment. Fortunately, these types of skin cancers are rare. When they do occur, they pose a significant reconstructive challenge, because they can expose cranium, dura, orbit, and sinus. Free tissue transfer has been a significant advance in the management of these tumors. It provides well-vascularized tissue that can withstand the detrimental effects of adjuvant radiation therapy as well as provide tissue with adequate volume not limited by arc of rotation. Most importantly, however, free tissue transfer allows an oncologist the ability to completely resect tumor without compromising surgical margins.
